# SB218078 inhibits angiogenesis and epithelial-mesenchymal transition in breast cancer

**DOI:** 10.3389/fphar.2025.1552707

**Published:** 2025-03-14

**Authors:** Qianxue Wu, Jiawei Xu, Xin Tang, Jin Yu, Benhua Li, Jun Yang, Xiang Zhang

**Affiliations:** ^1^ Department of Hepatobiliary Surgery, The First Affiliated Hospital of Chongqing Medical University, Chongqing, China; ^2^ Department of Endocrine and Breast Surgery, The First Affiliated Hospital of Chongqing Medical University, Chongqing, China; ^3^ Department of Respiratory and Critical Care Medicine, The First People’s Hospital of Chongqing Liang Jiang New Area, Chongqing, China; ^4^ Jiulongpo Center for Disease Control and Prevention of Chongqing, Chongqing, China; ^5^ Department of Clinical Laboratory, The Second People’s Hospital of Liangshan yi Autonomous Prefecture, Xichang, China; ^6^ Clinical Molecular Medicine Testing Center, The First Affiliated Hospital of Chongqing Medical University, Chongqing, China

**Keywords:** angiogenesis, ZEB1, breast cancer, EMT, SB218078

## Abstract

**Purpose:**

Small-molecule inhibitors of vascular endothelial growth factor receptor 2 (VEGFR2) face clinical limitations due to adverse effects. This study aimed to evaluate the novel compound SB218078 as a dual-targeting agent against both tumor angiogenesis and epithelial-mesenchymal transition (EMT) in breast cancer, while exploring its mechanisms of action.

**Methods:**

The anti-angiogenic effects of SB218078 were investigated using *in vitro* models of endothelial cell migration, invasion, and tube formation, alongside *in vivo* zebrafish developmental angiogenesis assays. Breast cancer progression was assessed through cellular proliferation, migration, invasion tests, and mouse xenograft models. Mechanistic studies focused on the Chk1/ZEB1 signaling axis, validated through genetic interventions.

**Results:**

SB218078 effectively suppressed angiogenesis by inhibiting endothelial cell functions and disrupting developmental vascular networks in zebrafish. It also impeded breast cancer cell aggressiveness and tumor growth *in vivo*. Mechanistically, SB218078 selectively targeted ZEB1—an EMT transcription factor—via Chk1 inhibition, with ZEB1 knockdown mimicking its anti-angiogenic effects, while ZEB1 overexpression reversed this activity.

**Conclusion:**

SB218078 emerges as a promising dual-action therapeutic candidate for breast cancer, simultaneously blocking angiogenesis and EMT through the Chk1-ZEB1 axis. Its specificity for ZEB1, distinct from other EMT regulators, offers a novel strategy to overcome the limitations of traditional VEGFR2 inhibitors, warranting further preclinical development.

## 1 Introduction

Breast cancer is one of the most common cancers worldwide and the most common cause of cancer death in women. Tumor angiogenesis is a critical process that supplies tumors with the necessary blood supply to facilitate growth and metastasis (Zhang et al., 2020). Tumor growth relies on blood vessels, which not only deliver oxygen and nutrients essential for tumor tissue but also serve as conduits for transporting tumor cells, thereby enabling metastasis (Shashni et al., 2021). However, breast cancer exemplifies a solid tumor type that has consistently shown limited responses to angiogenesis inhibitors, failing to significantly enhance patient survival outcomes (Ayoub et al., 2022). Consequently, the development of angiogenesis inhibitors targeting the blood vessels of breast cancer has become a vital focus in anti-tumor research.

Small molecule drugs inhibit tumor angiogenesis by specifically blocking the angiogenic signaling pathways. They are characterized by their non-immunogenic nature, ease of absorption, and various routes of administration (Li et al., 2021; Qin et al., 2019). Vascular endothelial growth factor receptor 2 (VEGFR2) is considered the most important target for anti-angiogenic therapy in breast cancer (Ash et al., 2021). Presently, a range of small molecule inhibitors of VEGFR2 is employed in clinical treatment, including FDA-approved tyrosine kinase inhibitors such as sorafenib (Fang et al., 2019), sunitinib (O'Donnell et al., 2015) and vandetanib (Middleton et al., 2017). Despite their effective anti-tumor activity, these drugs come with certain limitations and side effects. Notably, the incidence of cardiovascular toxicity related to VEGFR2-targeting tyrosine kinase inhibitors is relatively high, leading to potential adverse reactions in patients, such as heart failure and myocardial ischemia (Versmissen et al., 2019). Additionally, small molecule inhibitors like isoliquiritigenin (Wang et al., 2019), pazopanib (Grunwald et al., 2020), cediranib (Ledermann et al., 2016), and motesanib (Torok et al., 2017), have been tested in clinical trials. Observations during these trials revealed that patients often developed drug resistance following anti-angiogenic therapy (Liang et al., 2021; Malone et al., 2020). As a result, existing anti-angiogenic drugs have yielded only modest improvements in overall survival rates for cancer patients and are frequently associated with drug resistance. SB218078 is a compound that enhances the efficacy of certain chemotherapeutic drugs by inhibiting Chk1 activity, which prevents its phosphorylation of Cdc25 and releases G2 cell cycle arrest, thereby increasing the cytotoxicity of DNA-damaging agents. However, the role and mechanism of SB218078 in tumor anti-angiogenesis remain unclear.

In this study, we identified SB218078, a small molecule inhibitor that effectively suppresses tumor angiogenesis and epithelial-mesenchymal transition (EMT) in breast cancer. Using transgenic fluorescent zebrafish embryos Tg (Fli-1: EGFP) and human umbilical vein endothelial cells (HUVECs), we demonstrated that SB218078 significantly reduces tumor angiogenesis by inhibiting VEGF-induced phosphorylation of VEGFR2 and its downstream signaling pathways. Additionally, SB218078 markedly hinders the growth, migration, and metastasis of breast cancer cells in both *in vitro* and *in vivo* models. Mechanistically, it decreases the expression of EMT-related transcription factors, particularly downregulating zinc finger E-box binding homeobox 1 (ZEB1), which counteracts angiogenesis in zebrafish embryos. In summary, SB218078 is established as a potent anti-angiogenic agent, offering a promising new strategy for breast cancer treatment.

## 2 Methods

### 2.1 Animals

Transgenic zebrafish Tg (fli-1:EGFP) were used to study blood vessel development. Adult zebrafish were maintained under controlled conditions with a 14-hour light and 10-hour dark cycle to support their natural biorhythm. The breeding environment was kept at approximately 28.5°C, with a pH of 7.0 ± 0.2 and an electrical conductivity of 550 ± 10 μS/cm. Adult zebrafish, aged 7–18 months, were fed harvested shrimp twice daily and selected for spawning. Embryos were collected 1 hour post-spawning, washed in Holt Buffer to remove dead eggs and debris, and cultured at 28.5°C for development observation. Healthy embryos at 12 h post-fertilization (hpf) were transferred to a 24-well plate containing various concentrations of SB218078 (1.25 μM–10 μM) diluted in a solution with 1-phenyl 2-thiourea (PTU), while the negative control contained PTU and 0.1% dimethyl sulfoxide (DMSO). After 24 h of treatment, the embryos were examined under a fluorescence microscope for vascular development, specifically the intersegmental vessels (ISV) and dorsal longitudinal anastomotic vessels (DLAV), with abnormal cases recorded. For fin regeneration, zebrafish at 30 days post-fertilization (dpf) underwent dorsal fin amputation and were subsequently observed for vascular development and regeneration under a fluorescence microscope. They were maintained in a 12-well plate with a solution containing 10 μM SB218078, refreshed every 24 h, while their fin regeneration was monitored microscopically. In the tumor angiogenesis model, mCherry-labeled MDA-MB-231 cells were injected into the perivitelline space of 2-day-old zebrafish embryos, which were then treated with either DMSO (control) or 2.5 µM SB218078 for 3 days. Following treatment, embryos were fixed in 4% paraformaldehyde for imaging, and angiogenesis was analyzed using a spinning disk laser scanning confocal microscope. Tumor angiogenesis was quantified with ImageJ software based on vessel intensity relative to tumor cell intensity. Isogenic zebrafish with gene knockouts were used to observe subintestinal vessel (SIV) formation at approximately 96 hpf, with embryos fixed, stored, and imaged at a wavelength of 488 nm. SIV vasculature was quantified using ImageJ, and leading buds were counted (Ma et al., 2021).

For the xenograft experiments, female nude mice (4–6 weeks old, Charles River, Beijing, China) were used. One million MDA-MB-231 cells were resuspended in 100 μL of PBS and injected into the mammary fat pad of the mice in the groin area. The mice with tumors were randomly divided into two groups of six: the treatment group received an intragastric injection of 5 mg/kg/d of SB218077, while the control group was given an equivalent volume of vehicle. Tumor growth, body weight, and tumor size were monitored weekly, with tumor volume calculated using the formula V (mm³) = Length (mm) × Width (mm)^2^/2. For the Matrigel plug studies, mice were anesthetized with 2% isoflurane. A syringe pre-cooled to −20°C was used to absorb the Matrigel mixture, which was then injected into a 1-cm gap created subcutaneously in the armpit. The mice were routinely fed and monitored, with body weights recorded every 3 days. Ten days post-operation, the mice were sacrificed, and the Matrigel plugs were dissected for immunofluorescence using frozen sections. All animal experiments received approval from the Institutional Ethics Committees of the First Affiliated Hospital of Chongqing Medical University.

### 2.2 Cell culture

Fetal bovine serum (FBS), RPMI-1640 medium, Penicillin-Streptomycin solution, and 0.25% trypsin were all obtained from Invitrogen (Carlsbad, CA). Human umbilical vein endothelial cells (HUVECs), along with breast cancer cell lines T47D and MDA-MB-231, were obtained from ATCC. T47D and MDA-MB-231 cells were maintained in RPMI-1640 medium supplemented with 10% fetal bovine serum (FBS) and 1% Penicillin-Streptomycin solution. HUVECs were cultured in EGMTM-2 Bullet Kit Endothelial Cell Medium (Lonza, United States), along with 1% penicillin/streptomycin. The culture medium was refreshed every two to 3 days to ensure optimal growth conditions.

### 2.3 Rat arterial ring

The aorta isolated from a 6-week-old Sprague Dawley (SD) rat was sliced into thin sections approximately 1 mm thick and arranged in a 96-well plate. Each well was filled with 70 μL of Matrigel to embed the arterial rings, which were then incubated at 37°C for 1 h. Following this, different concentrations of SB218078 (1 μM and 2 μM) were introduced to each well for routine culture. After 7 days, microvascular sprouting from the arterial rings was observed under a microscope and documented through photography.

### 2.4 Migration assay

Approximately 2.5 × 10^5 cells per 2 mL per well (either HUVECs or MDA-MB-231 and T47D cells) were inoculated in 6-well plates. Once the cells reached 95% confluence, a wound was created in each well using a 10-µL pipette tip. Images were captured under a microscope at this initial time point (0 h). The drug SB218078 was diluted to various concentrations (1 μM and 2 µM), and 2 mL of each solution was added to the wells. After 24 h, the same visual field was examined, and images were obtained for analysis.

### 2.5 Transwell assay

The transwell chamber and its tips were precooled at −20°C. Next, 50 μL of melted Matrigel was added to the transwell chamber and incubated at 37°C for 1 h to allow it to solidify. Meanwhile, full medium (200 μL) was added to each well of a 24-well plate. The transwell chamber was then placed into the wells. Cells in the logarithmic growth phase (MDA-MB-231, T47D, and HUVECs) were collected and suspended in culture medium. A total of 2 × 10^4 cells in 500 μL were seeded into the transwell chamber and incubated at 37°C with 5% CO2 for 16 h. After this incubation, the chamber was removed, and the cells remaining in the chamber were gently wiped off. The chamber was then fixed with 4% paraformaldehyde (PFA) for 30 min and subsequently stained with crystal violet for 10 min. Images were captured using a microscope (Trinocular Biological Microscope, CX43, Olympus, Tokyo, Japan).

### 2.6 Tube formation

The 48-well plate and pipette tips were precooled at −20°C. Subsequently, 160 μL of melted Matrigel was added to the 48-well plate and incubated at 37°C for 1 h to allow it to solidify. HUVECs in the logarithmic growth phase were collected and suspended in treatment solutions (vehicle control (DMSO) or 1, 2 µM SB218078). A total of 4 × 10^4 HUVECs in 500 μL were then added to each well of the 48-well plate and cultured at 37°C with 5% CO2 for 6 h. Images were captured using a microscope (Trinocular Biological Microscope, CX43, Olympus, Tokyo, Japan).

### 2.7 Clone formation

Cells (HUVECs, T47D, and MDA-MB-231) in the logarithmic growth phase were collected and cultured in a 6-well plate at a density of 600 cells per 2 mL per well. The drug SB218078 was diluted to various concentrations (1 μM and 2 µM), and 2 mL of each concentration was added to each well. The drug-containing medium was changed every 48 h. After 1 week, the cells were washed twice with PBS and fixed with 4% paraformaldehyde (PFA) for 30 min. Subsequently, the cells were stained with crystal violet for 10 min at room temperature (RT). Manual counting was performed to determine the number of cell clones present (Wu et al., 2021).

### 2.8 Kinase assay

Different concentrations of SB218078 were employed to inhibit intracellular protein kinases. In brief, 10 µL of non-radioactive ATP solution (in H_2_O), 25 µL of an assay buffer/[γ-³^2^P]-ATP mixture, varying concentrations of SB218078 dissolved in 10% DMSO, and 10 µL of enzyme/substrate were combined in each well of a 96-well FlashPlates™. The reaction was carried out at 30°C for 60 min. All protein kinase assays were conducted using a Beckman Coulter Biomek 2000/SL robotic system (Ma et al., 2021).

### 2.9 Lentiviral transfection

The Biosettia website (http://biosettia.com/support/shrna-designer) was utilized to design shRNAs. The sequences of the shRNAs were as follows: sh-ZEB1#1, 5′-ATA​GAG​GCT​ACA​AGC​GCT​TTA-3′; sh-ZEB1#2, 5′-GGA​GGA​TAA​AGA​GAT​GGA​AGA-3′; sh-Snail#1, 5′-GCT​GTC​ACT​CAA​TTC​GGT​CAT​T-3′; sh-Snail#2, 5′-GCA​CCG​GCG​TTT​CAG​ATA​TCT-3′; sh-Twist#1, 5′-GGA​AGA​GGC​GAT​ATC​GGA​AGT-3′; and sh-Twist#2, 5′-GGA​TGG​AAG​GTG​CGT​GGA​ATA-3′. These shRNAs were cloned into the pLVX-shRNA1-puro vector to produce the pLV-shRNA vector. HEK293T cells were transfected with the lentiviral vector using Lipofectamine 2000, and the lentivirus was generated by transfecting a packaging plasmid via calcium phosphate and subsequently collecting the supernatant. When the breast cancer cell density reached 30%–50%, the cells were infected with a mixture of 1 mL of lentivirus stock and 3 µL of Polybrene (Solarbio, Beijing, China) at 37°C. After 48 h, stable cell lines were selected using puromycin for 48–72 h, after which follow-up experiments were conducted.

### 2.10 Immunohistochemistry (IHC)

The tissue slices (xenografts) were sectioned to a thickness of 4 μm and dewaxed using xylene. Antigen retrieval was performed using citrate buffer (pH 6.0): the slices were immersed in this buffer at 95°C for 20 min and then allowed to cool to room temperature. Following this, the slices were treated with a 3% peroxidase solution (ZSGB-BIO, Beijing, China) for 20 min to inhibit endogenous peroxidase activity. To block non-specific binding sites, 10% normal goat serum (ZSGB-BIO) was applied to the slices at 37°C for 30 min, after which the slices were incubated overnight at 4°C with the primary antibody (1:200 dilution). The following day, the slices were incubated with biotinylated goat anti-mouse IgG (ZSGB-BIO) for 30 min, washed, and then treated with horseradish peroxidase-labeled streptavidin-biotin (ZSGB-BIO) for 20 min. Finally, the slices were stained with DAB under a microscope, re-stained with hematoxylin, dehydrated, and mounted. The slices were examined under a microscope, and the intensity of staining alongside the percentage of immunoreactive cells were used to assess protein expression (Wu et al., 2022).

### 2.11 Cell viability assay

Approximately 3,000 cells from each group (HUVECs, T47D, and MDA-MB-231, treated with different concentrations of SB218078 for 72 h) were inoculated into 96-well culture plates. At 0, 24, 48, and 72 h, a mixture of 10 μL of CCK-8 reagent (CK04, Dojindo, Japan) and 100 μL of medium was added to each well and incubated with the cells for 2 h. The absorbance values were then measured at 450 nm using a microplate reader (Infinite 200 PRO, Tecan, Männedorf, Switzerland).

### 2.12 Apoptosis assay

Well-grown MDA-MB-231 cells were collected and seeded in 6-well plates at a density of 1 × 10^5 cells per 100 mL per well. The drug SB218078 was diluted to the desired concentrations (1 μM and 2 µM), and the cells were incubated with the drug solution for 72 h. Approximately 1 × 10^6 cells were then treated with Annexin V-FITC (Sigma-Aldrich) and Propidium Iodide (Sigma-Aldrich) at room temperature for 30 min. Cell apoptosis was subsequently assessed using a FACSCalibur flow cytometer (BD Biosciences).

### 2.13 Western blotting

Total protein was extracted from HUVECs or breast cancer cells using RIPA lysis buffer (P0013B, Beyotime), and the protein concentration was quantified using a BCA kit (P0012S, Beyotime). Equal amounts of denatured protein (40 μg) were subjected to protein imprinting, followed by 10% sodium dodecyl sulfate–polyacrylamide gel electrophoresis (SDS-PAGE), and transferred onto a PVDF membrane. The membrane was blocked with 5% skim milk. It was then incubated overnight at 4°C with primary antibodies (Anti-pVEGFR2, anti-VEGFR2, anti-pSTAT3, anti-STAT3, anti-p-ERK, anti-ERK, anti-p-mTOR, anti-mTOR, anti-ZEB1, anti-Snail, anti-Vimentin, anti-Twist, anti-E-cadherin, and anti-β-actin). The following day, the membrane was incubated with a secondary antibody (1:2000 dilution) at room temperature for 1 h. The bands were detected using a chemical developer (Kodak).

### 2.14 Immunofluorescence imaging

Approximately 1 × 10^4 MDA-MB-231 cells were seeded onto slides in 24-well plates. The drug SB218078 was diluted to the desired concentrations (1 μM and 2 µM), and the cells were incubated with the drug solution for 72 h. Following this, the slides were fixed with 4% paraformaldehyde (PFA) for 30 min. After blocking with 5% goat serum for 15 min, the cell climbing slides were incubated overnight at 4°C with primary antibodies (anti-Vimentin (#5741) and anti-E-cadherin (#14472)). The next day, the slices were washed with PBS and incubated for 3 h with goat anti-human IgG labeled with FITC or Cy3. After DAPI staining for 5 min, the slides were mounted using an anti-fluorescence quenching solution (P0126, Beyotime). The immunofluorescence staining procedure for frozen xenograft slices followed a similar protocol, using anti-CD31 antibodies. Immunofluorescence was analyzed using ImageJ version 1.52a.

### 2.15 Generation of isogenic zebrafish with individual gene knockouts

For guide RNA (gRNA) target site design, potential gRNA target sites were identified using the web programs CRISPR Design (http://CRISPR.mit.edu) or CHOPCHOP (http://chopchop.cbu.uib.no/index.php). The following single guide RNAs (sgRNAs) (sense strand) were used: snail1a:sg-snail1a: 5′-GCA​CAA​GTT​GCC​ATT​TGC​CG-3′, twist1a:sg-twist1a: 5′-GAC​GTC​CCA​GAC​CAG​TCC​GG-3′, zeb1a:sg-zeb1a: 5′-GCC​AGC​CGC​AAA​AAC​GCC​G-3′, and green fluorescent protein (GFP): sg-GFP 5′-GGG​CGA​GGA​GCT​GTT​CAC​CG-3′. The sgRNA: Cas9 ribonucleoprotein (RNP) complexes included 1 μL 25 μM sgRNA + 1 μL 25 μM Cas9 stock + 2 µL H_2_O+ 1 μL 0.25% phenol red solution. Prior to microinjection, the RNP complex solution was incubated at 37°C for 5 min. Approximately 1 nL 5 μM gRNA: Cas9 RNP complex was injected into the cytoplasm of one-cell stage embryos. Zebrafish mutants were screened and identified (Hoshijima et al., 2019). The primers used for knockout validation were as following: snail1a: PF:TTCCAGACTCACGCTGACATC, PR:GTCCCAGAAATGCAACGACG; Twist1a: PF: GTG​TTC​GCG​CTG​CAT​GTA​AA, PR: TAA​ACG​CTT​CCT​CGC​GGA​TT; zeb1a: PF: GTG​TTC​GCG​CTG​CAT​GTA​AA, PR: TAA​ACG​CTT​CCT​CGC​GGA​TT.

### 2.16 RNA-seq analysis

The MDA-MB-231 cells were treated with SB218078 (2 μM) for 48 h, after which they were collected for RNA sequencing. Gene Ontology (GO) and Kyoto Encyclopedia of Genes and Genomes (KEGG) enrichment analyses were conducted using R software. The criteria for identifying differentially expressed proteins included a false discovery rate (FDR) corrected p-value of less than 0.01 and a log fold change (logFC) greater than 1. The names of the repository/repositories and accession number(s) can be found below: https://bigd.big.ac.cn/gsa/browse/ HRA008767.

### 2.17 Statistical analyses

The Student’s t-test and one-way analysis of variance (ANOVA) were employed to perform differential comparisons between two groups or multiple groups, respectively. For group comparisons within one-way ANOVA, Tukey’s test was applied. R software was used to analyze and perform statistics on RNA sequencing data. Statistical analyses were conducted using GraphPad Prism (version 5.0). A p-value of less than 0.05 was considered statistically significant.

## 3 Results

### 3.1 Inhibitory effects of SB218078 on vascular development in zebrafish embryos

**FIGURE 1:**
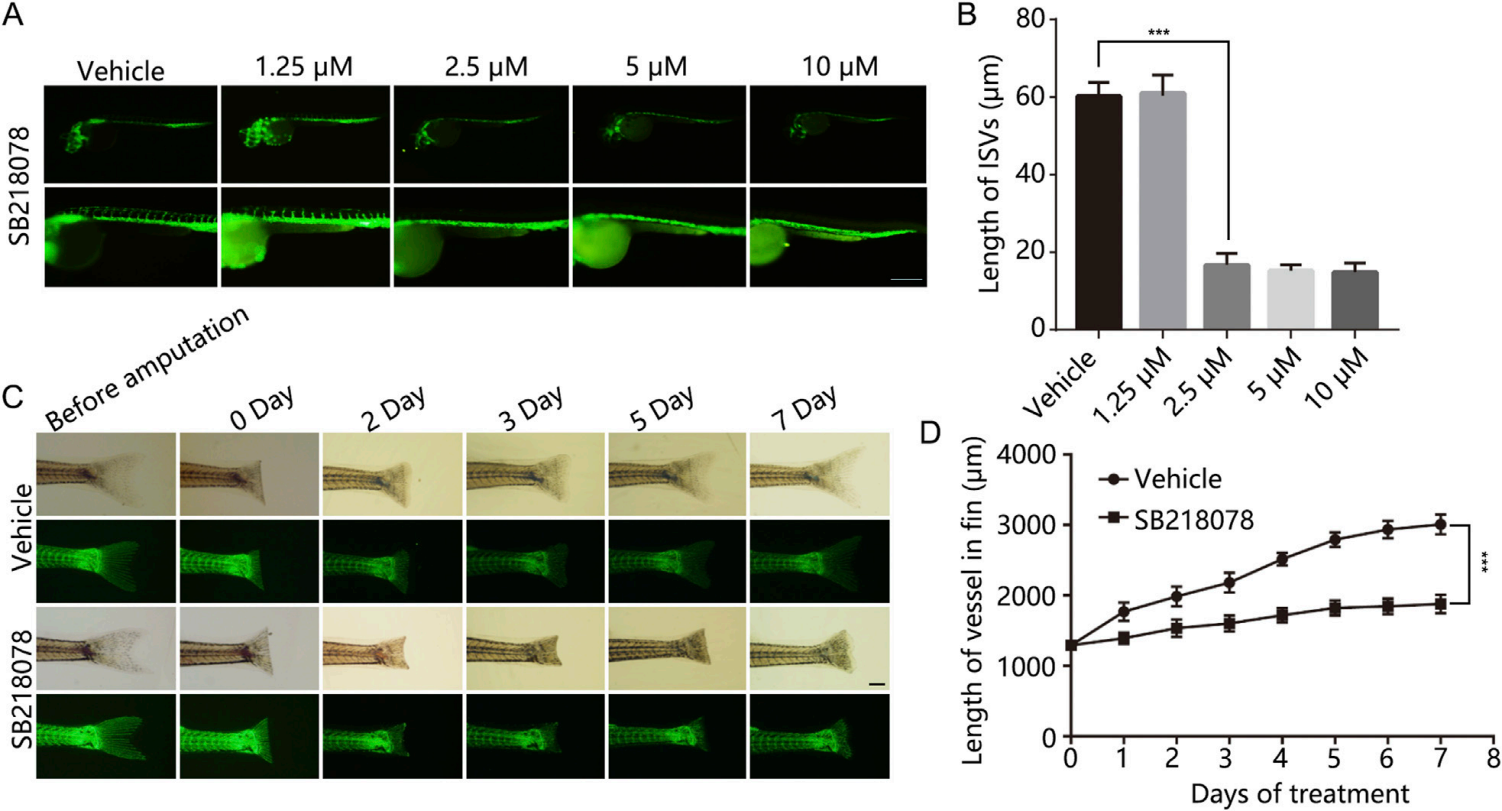
SB218078 Inhibits Vascular Development in Zebrafish Embryos. **(A, B)** Representative images of intersegmental vessels (ISVs) in zebrafish embryos at 12 h post-fertilization (hpf) treated with vehicle control and various concentrations of SB218078 (1.25 μM, 2.5 μM, 5 μM, 10 μM). **(C, D)** Regeneration of amputated zebrafish fins was monitored over a 7-day period. *P < 0.05, **P < 0.005, ***P < 0.001. Scale bar in **(A and C)** 50 μm.

We initially identified 30 candidate small-molecule drugs that could induce tumor cell death by targeting VEGFR2 activity using the molecular drug bank in our laboratory (see Table 1). To evaluate their effects on angiogenesis, we monitored the vascular development of intersegmental vessels (ISVs) and the dorsal longitudinal anastomotic vessel (DLAV) in zebrafish. At 12 h post-fertilization (hpf), zebrafish embryos were treated with small-molecule inhibitors for a duration of 24 h. The results revealed that SB218078, at a concentration of 2.5 μM, significantly inhibited the development of ISVs in zebrafish (Figures 1A, B). Given the importance of angiogenesis in zebrafish caudal fin regeneration (Ma et al., 2017), juvenile zebrafish (approximately 30 days post-fertilization) with severed tails were treated with 10 μM SB218078 and observed continuously for 7 days. We observed a significant reduction in both caudal fin regeneration and vascular growth in zebrafish following SB218078 treatment (Figures 1C, D), thereby confirming the inhibitory effect of SB218078 on angiogenesis in zebrafish.

**TABLE 1:** Thirty candidate small molecular drugs.

No.	Drugs	No.	Drugs
1	Meclizine hydrochloride	16	Phenylmer curic acetate
2	Fenbendazole	17	Ouabain
3	Vincristine sulfate	18	Pararosaniline pamoate
4	Proscillaridin	19	Mitoxantrone hydrochloride
5	Vinblastine sulfate	20	Sertraline hydrochloride
6	Candesartan cilextil	21	Doxorubicin
7	Bromocriptine mesylate	22	Itraconazole
8	Dexamethasone	23	SB 218078
9	Digitoxin	24	IMD 0354
10	Digoxin	25	SN 38
11	Emetine	26	(1′S, 2′S)-Nicotine-1′-oxide (1′S, 2′S)
12	Gentian violet	27	Cantharidin
13	Gramicidin	28	NSC-663284
14	Hexachlorophene	29	5-Iodotubercidin
15	Histamine dihydrochloride	30	Ivermectin

### 3.2 Inhibitory effects of SB218078 on angiogenesis in HUVECs

**FIGURE 2:**
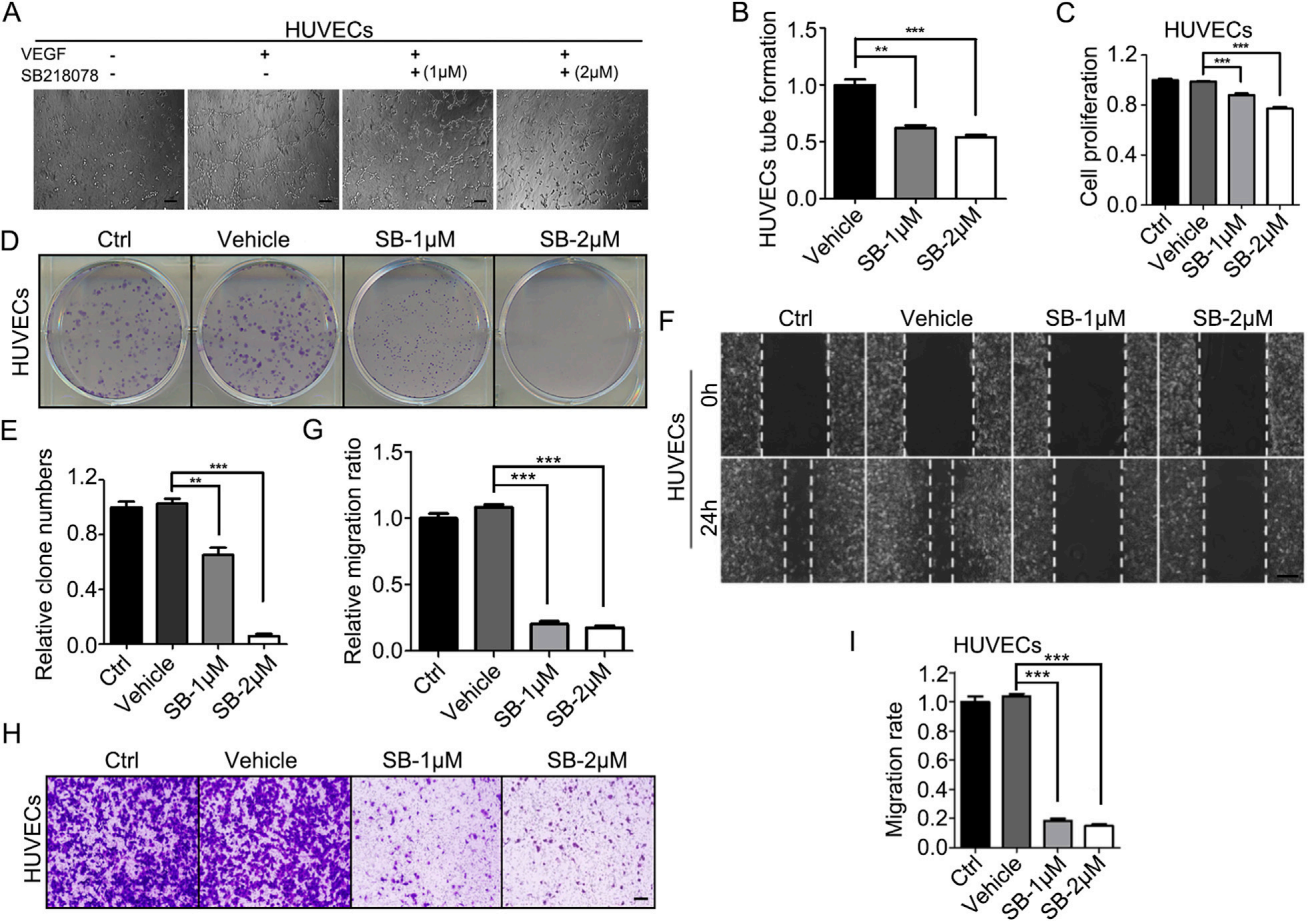
SB218078 inhibits angiogenesis *in vitro*. **(A, B)** SB218078 (1 μM and 2 μM)) inhibits VEGF-induced tube formation in human umbilical vein endothelial cells (HUVECs). **(C)** Treatment with SB218078 (1 μM and 2 μM) for 72 h significantly reduces HUVEC proliferation. **(D, F)** SB218078 (1 μM and 2 μM) inhibits the formation of HUVEC colonies. **(E, G–I)** SB218078 also inhibits HUVEC migration and invasion. Scale bar in **(A, F, and H)**: 50 μm.

Next, we utilized human umbilical vein endothelial cells (HUVECs) to assess the inhibitory effects of SB218078 on angiogenesis. HUVECs were cultured on Matrigel and treated with SB218078 in the presence of VEGF. As illustrated in Figure 2A, treatment with 1–2 μM SB218078 reduced the tube formation capability of HUVECs by approximately 50% compared to the vehicle control group (Figure 2B). We further investigated the impact of SB218078 on HUVEC proliferation using cell viability and colony formation assays. Our findings indicated that treatment with 1–2 μM SB218078 significantly inhibited the proliferation of HUVECs (Figures 2C–E). Additionally, wound-healing and transwell assays were conducted to evaluate the effects of SB218078 on HUVEC migration and invasion. The results demonstrated that SB218078 significantly inhibited both the migration (Figures 2F, G) and invasion (Figures 2H, I) of HUVECs *in vitro*.

**FIGURE 3:**
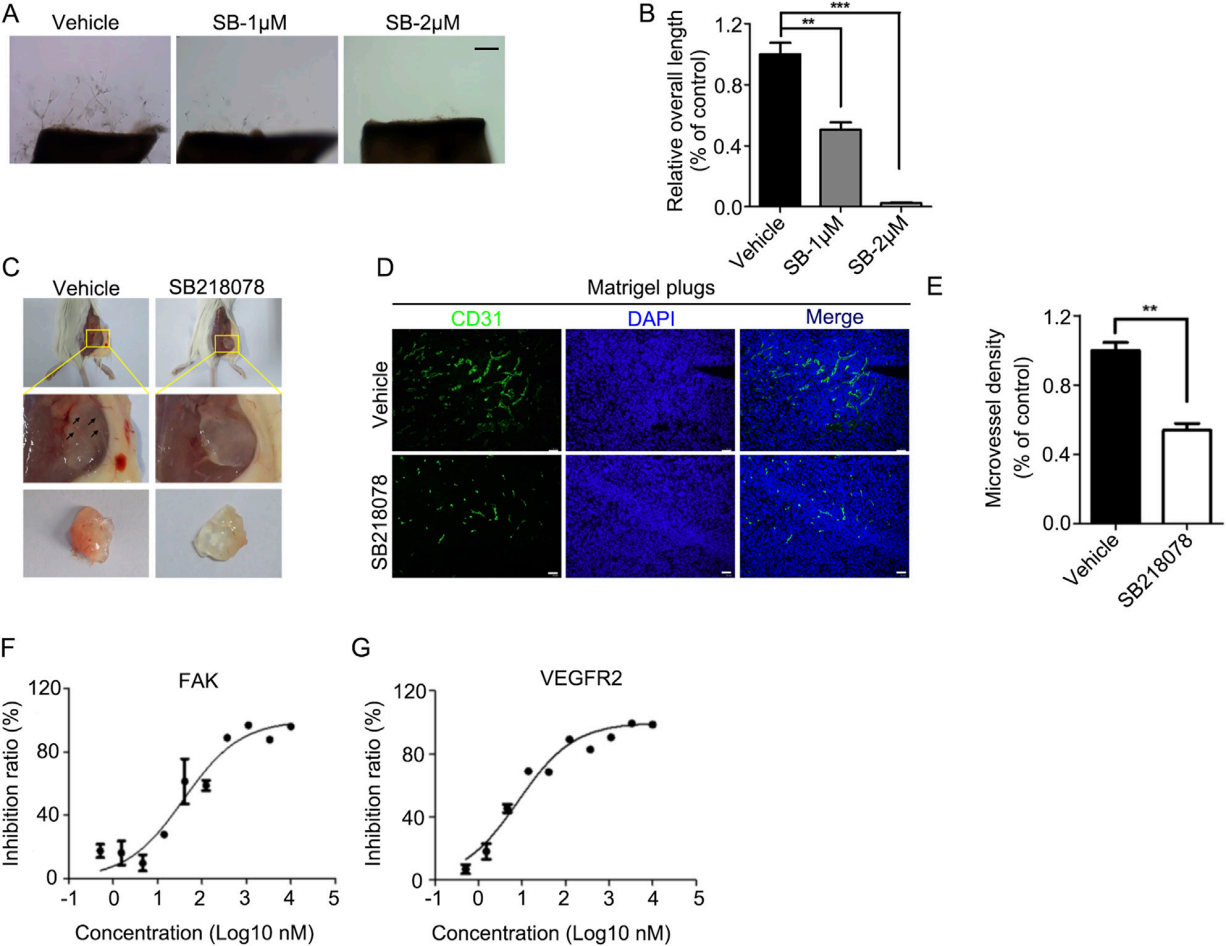
SB218078 inhibits angiogenesis *in vivo*. **(A)** Representative images showing the sprouting of vascular endothelial cells in different groups treated with vehicle control and SB218078 (1 μM and 2 μM) in the rat arterial ring assay. **(B)** Quantification of the relative overall length of endothelial cell sprouting induced by SB218078 in each treatment group. **(C)** Representative images of the Matrigel plug assay in mice treated with vehicle control (DMSO) and SB218078 (2 μM). **(D, E)** Immunofluorescence staining for CD31, along with statistical analysis of microvessel density. **(F, G)** Kinase assays conducted to identify potential targets of SB218078 for its anti-angiogenic effects. Scale bar in **(A, D)**: 50 μm.

For *in vivo* assessments, the aortic ring sprouting assay revealed that 2 μM SB218078 suppressed aortic ring sprouting by over 90% (Figures 3A, B). Furthermore, we established a subcutaneous Matrigel plug assay to evaluate the inhibitory effect of SB218078 on angiogenesis in mice. A 1 mL Matrigel mixture (comprising 750 μL Matrigel and 250 μL of endothelial medium containing 8 mg/kg SB218078 and 100 ng/kg VEGF) was injected subcutaneously into the axillae of BALB/c mice. Ten days later, the Matrigel plug was excised, and angiogenesis was assessed. As shown in Figure 3C, treatment with SB218078 significantly inhibited angiogenesis in mice. We also collected the Matrigel plugs for frozen sectioning and analyzed the expression of CD31, a marker for vascular endothelial cells, using immunofluorescence. The results indicated that the number of vascular endothelial cells migrating into the Matrigel plugs was reduced by 50% in the SB218078 treatment group (Figures 3D, E).

### 3.3 Molecular mechanisms underlying the angiogenesis inhibition effects of SB218078

To further elucidate the potential signaling pathways through which SB218078 exerts its anti-angiogenic effects, we conducted kinase activity analysis to determine the IC50 values of SB218078 on angiogenesis-related signaling pathway proteins (Table 2). The results revealed that the concentrations required to achieve 50% inhibition of FAK and KDR (VEGFR2) were notably low, particularly for VEGFR2, which demonstrated an especially low IC50 (Figures 3F, G; Table 2).

**TABLE 2:** The IC50 of SB218078 on angiogenesis-related signaling pathway proteins.

Kinase	IC50 (nM)
KDR	6.1
ERK1	>10,000
ERK2	>10,000
PKCα	>10,000
AKT	>10,000
FAK	49

### 3.4 SB218078 suppresses breast cancer cell growth and tumor angiogenesis *in vitro* and *in vivo*

**FIGURE 4:**
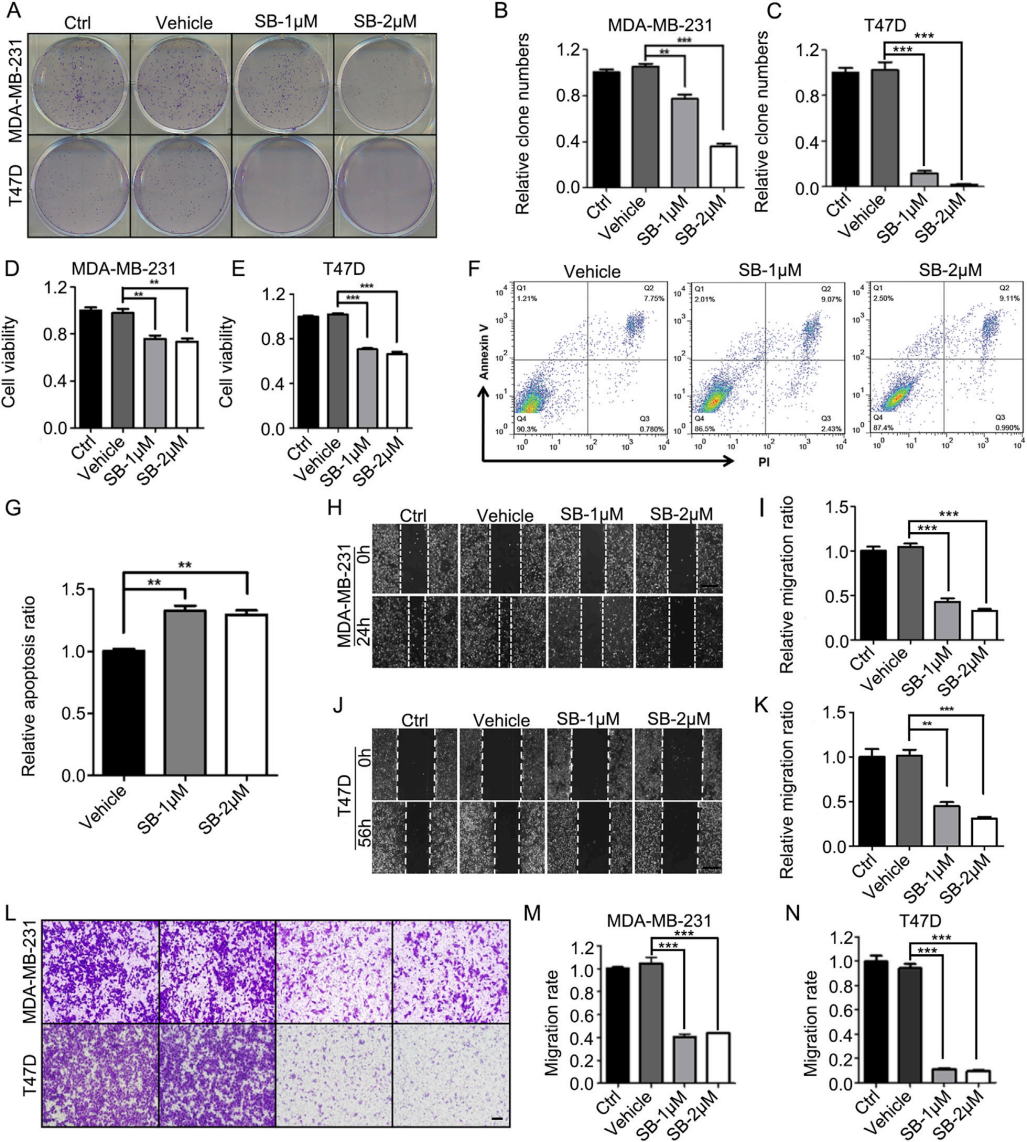
SB218078 inhibits the growth of breast cancer cells *in vitro*. **(A–C)** SB218078 (1 μM and 2 μM) significantly inhibits colony formation in T47D and MDA-MB-231 cells. **(D, E)** Treatment with SB218078 (1 μM and 2 μM) for 72 h reduces the proliferation of T47D and MDA-MB-231 cells. **(F, G)** Treatment with SB218078 (1 μM and 2 μM) for 72 h promotes the cell apoptosis of T47D and MDA-MB-231 cells. **(H–N)** SB218078 (1 μM and 2 μM) also inhibits the migration and invasion of both T47D and MDA-MB-231 cells. Scale bar in **(H, J, and L)**: 50 μm.

Considering the critical role of angiogenesis-related signaling pathways in the growth and metastasis of breast cancer, SB218078 may have significant potential as a therapeutic agent against this disease. To test this hypothesis, we examined the effects of SB218078 on the growth of T47D and MDA-MB-231 breast cancer cells. The results from colony formation (Figures 4A–C) and cell viability (Figures 4D, E) assays demonstrated that treatment with 1–2 μM SB218078 effectively inhibited the growth of breast cancer cells. Additionally, we investigated cancer cell apoptosis and found that SB218078 induced apoptosis in MDA-MB-231 cells (Figures 4F, G). Concurrently, wound-healing and transwell assays revealed that SB218078 significantly inhibited both the migration and invasion of breast cancer cells (Figures 4H–N).

**FIGURE 5:**
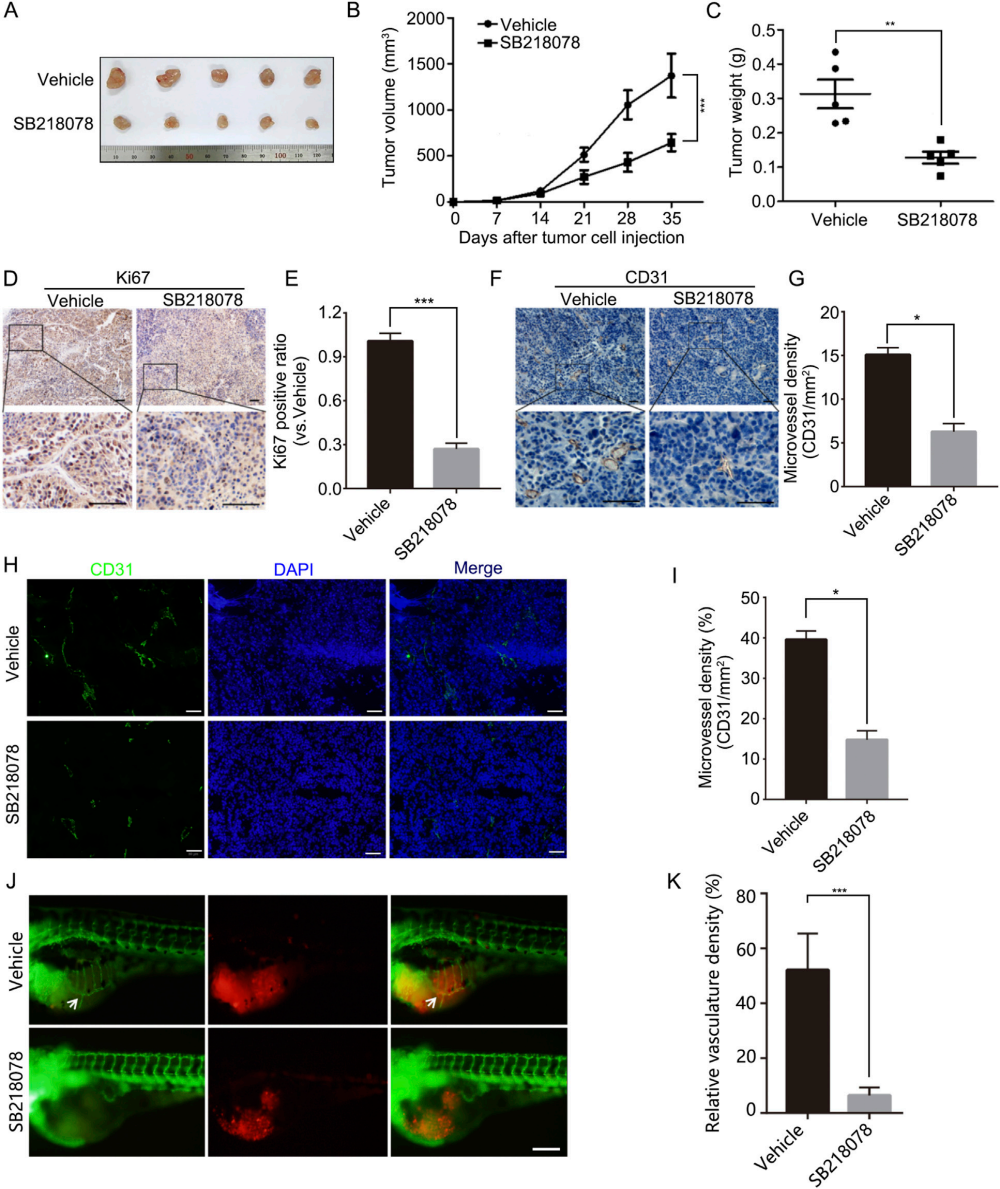
SB218078 inhibits the growth and angiogenesis of breast cancer *in vivo*. **(A)** Representative images of MDA-MB-231 cell xenografts treated with either vehicle control (DMSO) or SB218078. **(B)** Growth curve showing the progression of the xenografts, and **(C)** the corresponding weights of the xenografts. **(D, E)** Immunohistochemical (IHC) staining for Ki67 in xenograft tissues. **(F, G)** IHC analysis of microvessel density (CD31/mm²) between the two treatment groups in xenograft tissues. **(H, I)** Immunofluorescence assessment of microvessel density (CD31/mm^2^) in xenograft tissues for both groups. **(J)** Representative images depicting eGFP-expressing blood vessels (green) and mCherry-labeled MDA-MB-231 cells (red) following treatment of zebrafish embryos with vehicle control (DMSO) or SB218078 (2.5 μM) for 3 days. Scale bars: 200 µm. (K) Quantification of relative vascular density in each group. Scale bar in **(D, F, H, and J)** 50 μm.

To further assess the inhibitory effects of SB218078 on breast cancer cell growth, we established xenograft models using nude mice. The results indicated that intraperitoneal injections of SB218078 markedly suppressed the growth of transplanted tumors in these mice (Figures 5A–C). Furthermore, immunohistochemistry (IHC) analysis of the tumor tissue confirmed that the expression of Ki-67 was significantly reduced in response to SB218078 treatment (Figures 5D, E). Additionally, the transplanted tumor tissues were stained with CD31 using both IHC and immunofluorescence (IF). The results showed a significant decrease in the expression level of CD31 and microvessel density (MVD) following SB218078 treatment (Figures 5F–I). To further validate our findings, mCherry-labeled MDA-MB-231 cells were injected into the perivitelline space of zebrafish embryos. Interestingly, we observed that SB218078 significantly inhibited vascular formation surrounding the injected tumor cells (Figures 5J, K), confirming that SB218078 effectively suppresses tumor-related angiogenesis *in vivo*.

### 3.5 ZEB1-independent effects of SB218078 on angiogenesis

**FIGURE 6:**
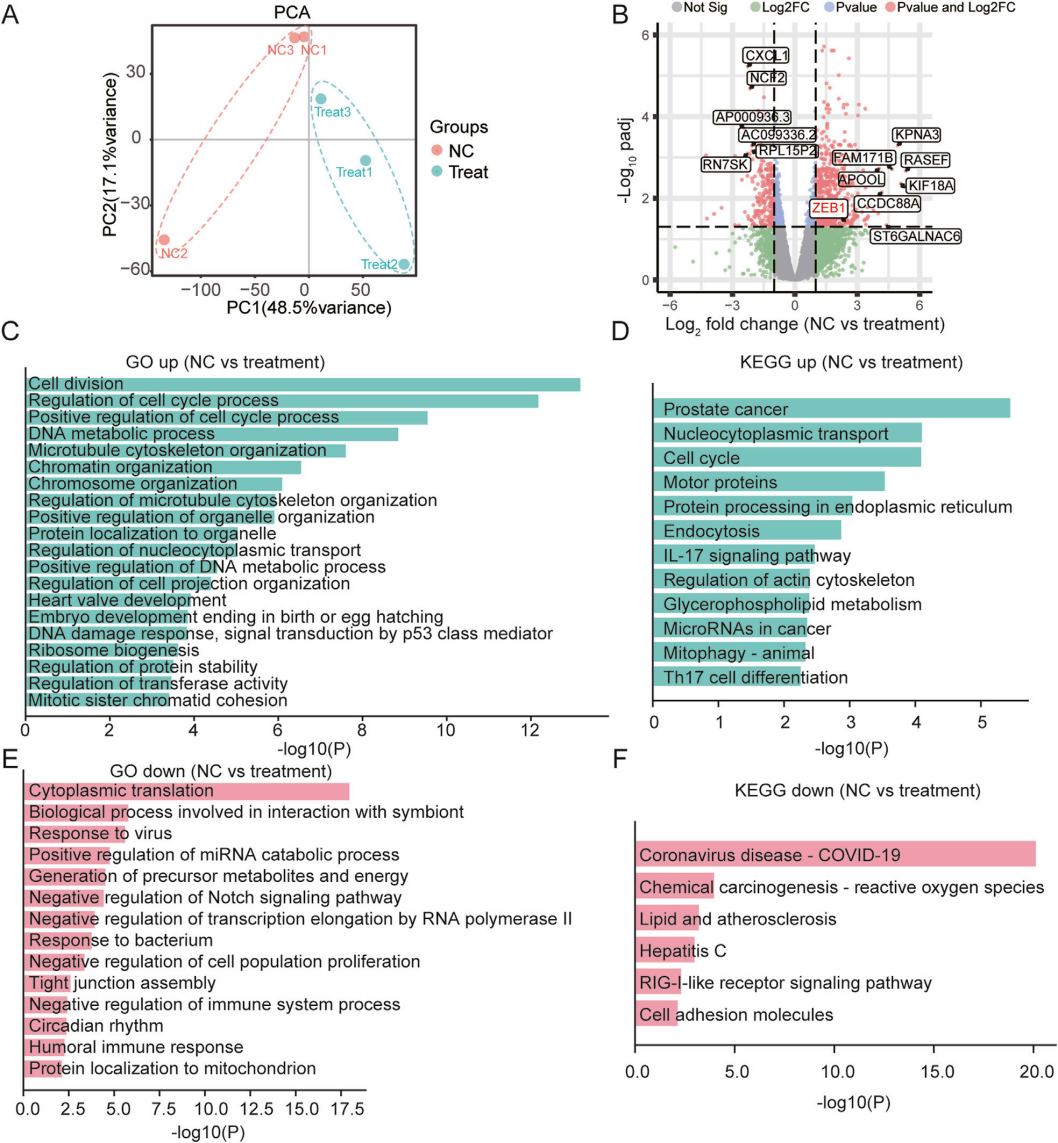
SB218078 Inhibits the EMT in Breast Cancer Cells. **(A)** RNA sequencing analysis of MDA-MB-231 cells treated with SB218078. **(B)** Volcano plot displaying the differentially expressed genes between the vehicle control and SB218078 treatment groups. **(C–F)** Gene enrichment analysis indicating that SB218078 regulates various biological processes, including the cell cycle, nuclear transcription factors, and tumor-related pathways, among others.

**FIGURE 7:**
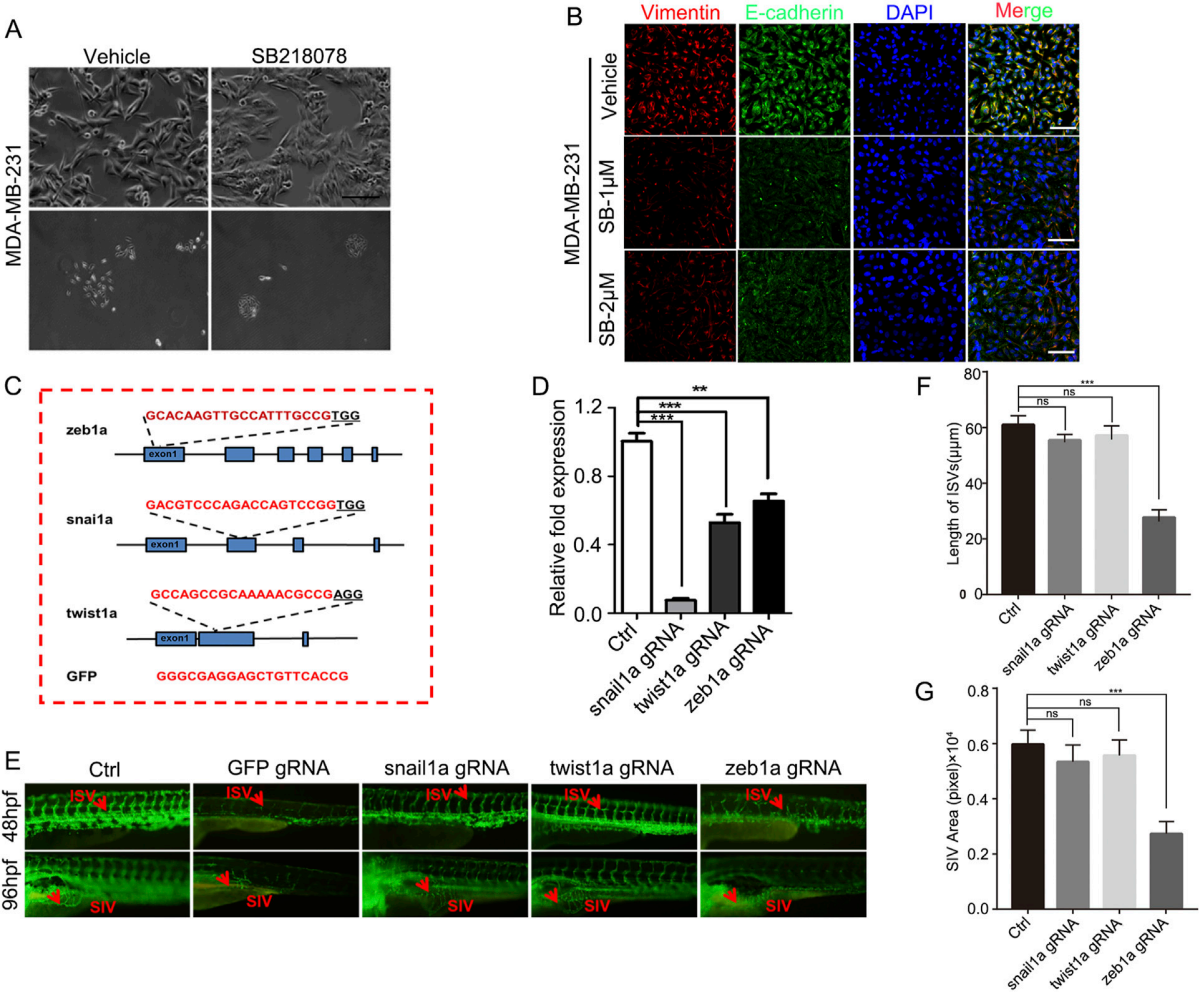
ZEB1-Independent Effects of SB218078 on Angiogenesis. **(A)** SB218078 inhibits EMT-related morphological changes in MDA-MB-231 cells. **(B)** Immunofluorescence analysis of protein levels of Vimentin and E-cadherin in MDA-MB-231 cells treated with either vehicle control (DMSO) or SB218078 (1 μM and 2 μM). **(C)** Schematic representation of the designed gRNA sequences for zeb1a, snail1a, twist1a, and GFP. **(D)** RNA expression levels of each target in zebrafish across different treatment groups. **(E–G)** Knockout of ZEB1 using the CRISPR system inhibits the vascular development of intersegmental vessels (ISVs) and subintestinal vessels (SIVs) in zebrafish.

To investigate the mechanism through which SB218078 inhibits angiogenesis in breast cancer, RNA sequencing was conducted on breast cancer cells treated with SB218078 (Figure 6A). The findings indicated that the well-known epithelial-mesenchymal transition (EMT) marker ZEB1 is influenced by SB218078 (Figure 6B). Gene enrichment analysis further revealed that SB218078 regulates various biological processes, including the cell cycle, nuclear transcription factors, and tumor-associated pathways (Figures 6C–F). Previous studies have identified Chk1, a target of SB218078, as a downstream protein of the EMT transcription factor ZEB1. Consistent with this, our results demonstrated that treatment with SB218078 significantly inhibited the EMT process in MDA-MB-231 cells (Figure 7A). Additionally, we observed a marked reduction in the expression of vimentin, a mesenchymal marker, in MDA-MB-231 cells treated with 1–2 μM of SB218078 (Figure 7B). In the *in vivo* assays, we employed the CRISPR/Cas9 system to silence the expression of ZEB1, Snail, and Twist in zebrafish (Figures 7C, D). The results revealed that specific targeting of ZEB1, as opposed to Snail or Twist, significantly inhibited the development of intersegmental blood vessels (ISV) and subintestinal vessels (SIV) in zebrafish (Figures 7E–G). This demonstrates the EMT-independent effect of SB218078 on tumor angiogenesis *in vivo*.

## 4 Discussion

Anti-angiogenesis therapy has emerged as a promising strategy for treating malignant solid tumors (Tang et al., 2021). In this study, we identified SB218078, a small molecule originally developed as a Chk1 inhibitor, which acts as an angiogenesis inhibitor. This compound was designed to mitigate G2 phase arrest caused by topoisomerase I-induced DNA damage, thereby enhancing tumor cell apoptosis and overcoming drug resistance synergistically (Jin et al., 2005; Wang et al., 2008). Our findings build on previous research by demonstrating that SB218078 significantly inhibits the proliferation, migration, invasion, and angiogenesis of vascular endothelial cells, as well as the VEGF-induced activation of VEGFR2. Notably, SB218078 also exhibits strong inhibitory effects on the growth, migration, invasion, and angiogenesis of breast cancer cells both *in vivo* and *in vitro*. Therefore, our data suggest that SB218078 has considerable potential as a treatment for breast cancer due to its anti-tumor and anti-angiogenesis properties.

The developmental patterns of intersegmental vessels (ISVs) and dorsal longitudinal anterior vessels (DLAVs) in zebrafish align with those observed in tumor-related angiogenesis (Buglak et al., 2020; Wilkinson and van Eeden, 2014). Consequently, we employed the zebrafish embryonic vascular development model to assess the anti-angiogenic effects of SB218078. Our results demonstrated that treatment with 1–2 μM SB218078 significantly inhibited the development of subintestinal vessels (SIVs) and DLAVs. Additionally, we found that SB218078 effectively suppressed the proliferation, migration, invasion, and angiogenesis of vascular endothelial cells *in vitro*. However, the regulatory mechanisms underlying tumor angiogenesis are complex. VEGFR2, as a key regulator of vascular endothelial cell function, mediates cell proliferation through the MAPK/ERK signaling cascade (Lu et al., 2021a; Walker et al., 2021; Hultgren et al., 2020). Similar to these studies, our study showed that SB218078 could significantly inhibit the activation of VEGFR2.

In addition to inhibiting angiogenesis in vascular endothelial cells, SB218078 also reduced cell growth, migration, and invasion, while promoting apoptosis in breast cancer cells. As a Chk1 inhibitor, SB218078 induces apoptosis through mechanisms involving DNA damage and S-phase arrest, and it may enhance the efficacy of chemotherapeutic agents by acting as a sensitizer. Supporting this notion, our flow cytometry analysis indicated that SB218078 promoted apoptosis in breast cancer cells. Notably, our results also confirmed that SB218078 can inhibit the migration and invasion of tumor cells. While Chk1 primarily plays a role in DNA damage repair and homologous recombination, the upstream factor ZEB1 is a critical transcription factor that promotes EMT (Drapela et al., 2020). EMT is a significant biological mechanism underlying tumor invasion and metastasis (Zheng et al., 2015). Our findings demonstrated that SB218078 inhibited the activation of EMT-related transcription factors such as ZEB1, Snail, and Twist. To evaluate the relationship between EMT and angiogenesis, we silenced these transcription factors and discovered that ZEB1 specifically inhibited angiogenesis in zebrafish, whereas Snail and Twist did not. Consistently, a recent study showed that deletion of endothelial ZEB1 in tumor-bearing mice reduced tumor angiogenesis and led to sustained normalization of tumor vasculature by epigenetically repressing TGF-β signaling (Fu et al., 2020). Our previous research also elucidated the role of ZEB1 in tumor angiogenesis (Liu et al., 2016; Jiang et al., 2020). Therefore, in studying the effects of SB218078 on tumor angiogenesis, we also examined its impact on ZEB1, yielding promising results. Additionally, targeting ZEB1 has been shown to reduce bevacizumab-resistant glioma phenotypes (Chandra et al., 2020). Our RNA sequencing results showed that the RNA expression of ZEB1 was regulated by SB218078. Based on the fact that SB218078 is designed as a competitive binding inhibitor against chk1, we inferred that SB218078 could inhibit tumor angiogenesis by regulating the transcription of ZEB1 and thereby inhibiting the expression of VEGFR2. Given these insights, it is reasonable to propose that SB218078 may have multiple targets, including VEGFR2 and ZEB1, in addition to Chk1. Importantly, SB218078 has demonstrated the ability to inhibit tumor growth and angiogenesis *in vivo* in breast cancer models. Collectively, these findings suggest that SB218078 has significant potential as a novel anti-tumor angiogenesis drug.

The limitations of our research are as follows. Firstly, the anti-angiogenic effects of SB218078 have yet to be validated in a diverse range of tumor models. Secondly, pharmacokinetic assessments could be enhanced to better evaluate the metabolic characteristics of SB218078 *in vivo*. Lastly, the specific targeting mechanism of SB218078 on ZEB1 and VEGFR2 is still not clear enough. We will design experiments to clarify it in detail in the future.

## 5 Conclusion

In summary, our research demonstrated that, in addition to its role as a classic Chk1 inhibitor, SB218078 significantly inhibits angiogenesis, as well as the growth, migration, and invasion of breast cancer cells. Mechanistically, we found that SB218078 suppresses tumor angiogenesis by blocking the activation of VEGFR2 and ZEB1. Therefore, SB218078 holds promise as a novel therapeutic strategy for targeting tumor angiogenesis in breast cancer.

## Data Availability

The datasets presented in this study can be found in online repositories. The names of the repository/repositories and accession number(s) can be found in the article/supplementary material.
